# Kinetic Analysis of Label-Free Microscale Collagen Gel Contraction Using Machine Learning-Aided Image Analysis

**DOI:** 10.3389/fbioe.2020.582602

**Published:** 2020-09-22

**Authors:** Cameron Yamanishi, Eric Parigoris, Shuichi Takayama

**Affiliations:** ^1^Wallace H. Coulter Department of Biomedical Engineering, Georgia Institute of Technology, Atlanta, GA, United States; ^2^The Parker H. Petit Institute of Bioengineering and Bioscience, Georgia Institute of Technology, Atlanta, GA, United States

**Keywords:** pulmonary fibrosis, collagen contraction, fibroblasts, phenotypic assay, aqueous two-phase systems, machine learning

## Abstract

Pulmonary fibrosis is a deadly lung disease, wherein normal lung tissue is progressively replaced with fibrotic scar tissue. An aspect of this process can be recreated *in vitro* by embedding fibroblasts into a collagen matrix and providing a fibrotic stimulus. This work expands upon a previously described method to print microscale cell-laden collagen gels and combines it with live cell imaging and automated image analysis to enable high-throughput analysis of the kinetics of cell-mediated contraction of this collagen matrix. The image analysis method utilizes a plugin for FIJI, built around Waikato Environment for Knowledge Analysis (WEKA) Segmentation. After cross-validation of this automated image analysis with manual shape tracing, the assay was applied to primary human lung fibroblasts including cells isolated from idiopathic pulmonary fibrosis patients. In the absence of any exogenous stimuli, the analysis showed significantly faster and more extensive contraction of the diseased cells compared to the healthy ones. Upon stimulation with transforming growth factor beta 1 (TGF-β1), fibroblasts from the healthy donor showed significantly more contraction throughout the observation period while differences in the response of diseased cells was subtle and could only be detected during a smaller window of time. Finally, dose-response curves for the inhibition of collagen gel contraction were determined for 3 small molecules including the only 2 FDA-approved drugs for idiopathic pulmonary fibrosis.

## Introduction

Pulmonary fibrosis is a deadly lung disease, characterized by an aberrant wound healing response (Ahluwalia et al., 2014). Healthy lung parenchyma is progressively replaced with fibrotic scar tissue, reducing patients’ lung capacity and often leading to death. Although progress has been made in understanding disease mechanisms, treatment options are limited to merely slowing the decline of lung function (Maher and Strek, 2019). Part of the difficulty in studying pulmonary fibrosis arises from the complex interplay between different cell types, mechanics, genetics, and the microenvironment (Ahluwalia et al., 2014; Barkauskas and Noble, 2014; Borensztajn et al., 2014; Betensley et al., 2016). Phenotypic assays, which can measure more holistic responses than gene or protein expression assays, are an important, complementary set of tools to understand cell and tissue processes (Yamanishi et al., 2019).

**FIGURE 1:**
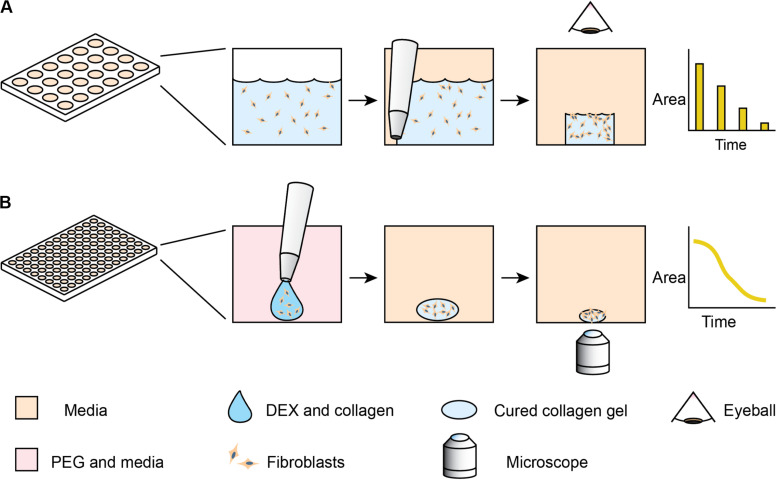
Comparison of standard collagen contraction assay with high-throughput ATPS method presented here. **(A)** Overview of traditional collagen contraction assay in a 24-well plate. After seeding fibroblasts in a collagen matrix, the gels are individually detached from the well and imaged at discrete time intervals. **(B)** In the ATPS collagen contraction assay, DEX and collagen droplets containing fibroblasts are mixed with PEG and media in 96-well plates. This creates a microgel in the center of the well, thereby eliminating the need for individual gel removal from the wall. The PEG and DEX solutions are then washed out, and then imaged every 2 h with an Incucyte microscope.

One of the classic phenotypic assays for pulmonary fibrosis is the collagen gel contraction assay (Bell et al., 1979). In this assay, fibroblasts are embedded into a collagen gel, which is detached from the surface of its container – usually a microplate well. Activated fibroblasts remodel the collagen gel, macroscopically shrinking the gel in a process similar to wound closure. Despite the assay’s utility and reliability in cell lines, the behavior of primary lung fibroblasts can be more subtle and difficult to detect (Campbell et al., 2012; Cui et al., 2016; Jin et al., 2019). These differences arise, as primary cells have variable initial states and sensitivities to stimulation. Primary cells present additional challenges, as they have limited growth capacity. Furthermore, the throughput has previously been low, as the collagen gel contraction assay traditionally requires the user to manually detach each gel from the edges of the well with a pipet tip (Bell et al., 1979). Measurement of the contracting area has also been manual, with pictures taken daily and images traced by hand (Figure 1A). While some collagen contraction assays have been adapted to a 96-well format (Kondo et al., 2004; Mohan and Bargagna-Mohan, 2016; Zhang et al., 2019), these higher-throughput assays have not been universally adopted due to challenges of manual detachment and image analysis. While several techniques have been developed for automated segmentation of label-free spheroid images (Rodday et al., 2011; Chen et al., 2014), these multicellular structures generally have high contrast compared to the media; cell-laden hydrogels have lower contrast, thereby requiring more modern image analysis algorithms.

To address these issues, we explore methods to increase the throughput of the assay and incorporate high frequency imaging. Our lab has previously developed a high-throughput collagen microgel bioprinting technique that does not require manual gel detachment and demonstrated its effectiveness with cell lines (Moraes et al., 2013). In this assay, evaporation of the small microgel during the gelation process is prevented by mixing the collagen and cells with an aqueous solution of dextran (DEX), which forms an aqueous two-phase system (ATPS) with an aqueous solution of polyethylene glycol (PEG). Collagen mostly remains within the DEX phase as it gels (Singh and Tavana, 2018), while the PEG phase provides an aqueous buffer, containing the collagen and limiting evaporation. This approach may be applicable to a variety of other hydrogel systems, however, our work here focuses on collagen. In this study, we extend the ATPS collagen bioprinting technique through automated imaging and image analysis (Figure 1B) to examine contraction kinetics of normal vs. diseased primary human lung fibroblasts, particularly in the context of anti-fibrotic drugs. Furthermore, this high frequency of sampling aids in distinguishing the more subtle differences in primary cells, compared to the cell lines used in our initial proof of concept study (Moraes et al., 2013).

## Materials and Methods

### Cell Culture

Normal human lung fibroblasts (NHLF, lot 0000655309, 56 year-old male) and idiopathic pulmonary fibrosis human lung fibroblasts (IPF, lot 0000627840, 52 year-old male) were purchased from Lonza (Walkersville, MD). These primary cells were cultured in complete Fibroblast Growth Medium (FGM-2, Lonza) and used from passages 2–5. For collagen gel contraction assays, cells were passaged into FGM-2, without serum (FGM-SF), then seeded into collagen gels the following day where they were collected at ∼75% confluence.

### Collagen Microgel Contraction Assay

Collagen microgel contraction assays were seeded as previously described (Moraes et al., 2013). 96-well round bottom microplates were filled with 100 μL per well of 6% (w/w) PEG, MW 35,000 (Sigma) dissolved in serum-free DMEM (Gibco) with 10% distilled water (Gibco) to adjust for osmotic pressure. This plate was warmed to 37°C in a 5% CO_2_ incubator. A collagen-dextran mixture was prepared on ice, consisting of 6% (w/w) DEX T500 (Sigma), 2 mg/mL Type I bovine skin collagen (Advanced Biomatrix), and 5 mM NaOH (Sigma) to neutralize the collagen. This mixture was mixed by pipetting up and down on ice, with care taken to avoid introducing bubbles. The mixture was kept on ice while cells were prepared for seeding. Cells were washed with PBS (Gibco), then trypsinized with 0.05% Trypsin (Gibco). After the cells lifted, they were quickly diluted in FGM-SF and centrifuged at 200 × g, 5 min, room temperature. After aspirating the supernatant, cells were resuspended in 1 mL FGM-SF and counted. Appropriate volumes of resuspended cells were centrifuged again and resuspended in DMEM. The cells were then mixed 1:1 with the collagen mixture to generate a 1 mg/mL collagen, 3% DEX solution. The collagen-DEX-cell suspension was transferred to a 96-well plate for seeding, where the DEX-cell suspension would be seeded into the wells containing PEG, as in Figure 1B.

### Liquid Handling and Imaging

As in our previous publication (Moraes et al., 2013), a Cybio FeliX liquid handler (Analytik Jena) was used to prepare collagen microgel plates (see Supplementary File for liquid handler script).

Collagen microgels were incubated and imaged using an Incucyte S3 (Sartorious) in-incubator microscope system. The Incucyte S3 performs auto-focus on each well of the microplate. 4x brightfield images were acquired at 1 h intervals for 2 days, then at 6 h intervals for the next 6 days.

### Drug Response Studies

Cell-laden collagen microgels were stimulated with or without 10 ng/mL TGF-β1 (R&D Systems) and anti-fibrotic drugs: nintedanib (Selleck Chem), pirfenidone (Selleck Chem), and the focal adhesion kinase inhibitor PF 431396 (Tocris) at specified concentrations. These collagen microgels were imaged over 8 days to monitor contraction. To determine the half maximal inhibitory concentration (IC50), the area under the curve (AUC) of the area over time graph was calculated for each individual gel as a parameter of overall contractility. These values were normalized, such that a gel with no contraction would have a normalized AUC of 100%. These contraction responses were fit to sigmoidal curves using the scipy module in python (Virtanen et al., 2020), using Eq. 1:

(1) $$R\,e\,s\,p\,o\,n\,s\,e=A+\frac{100-A}{1+10^{\left(\log\left(x\right)-\log\left(C\right)\right)*B}}$$

where A is the extent of contraction in the control condition, B is the Hill Coefficient, and C is the IC50.

### Image Processing

Collagen microgel areas were quantified using three methods: manual, Incucyte, and trainable WEKA segmentation. For manual quantification, gel perimeters were traced using ImageJ and areas were measured. For Incucyte quantification, images were segmented using the built-in Incucyte 2019A segmentation software from the Spheroid Module. In the trainable WEKA segmentation plugin from FIJI (ImageJ) (Arganda-Carreras et al., 2017), 1–10 representative microgel images were annotated and used to train the classifier. We wrote a new plugin (see Supplementary File) to iterate through a folder of images exported from the Incucyte 2019A software, apply the WEKA classifier, run quality checks, measure areas, and generate a.csv file containing areas, well positions, and times. Further analyses were performed using the pandas module in python (McKinney, 2010).

### Statistical Analysis

For the comparison between manual and algorithm measurements of gel areas, the Pearsons correlation coefficient was calculated. The differences between gel contraction for +/− TGF-β1 conditions at the indicated time points were analyzed using multiple *t*-tests with a Bonferroni correction and a 95% confidence interval. Lastly, standard deviations for parameter estimates of the IC50 values in the drug dose response studies were acquired from the covariance matrix of the model fit (scipy.optimize.curve_fit module in Python).

## Results

### Validation and Optimization of Machine Learning Image Processing

**FIGURE 2:**
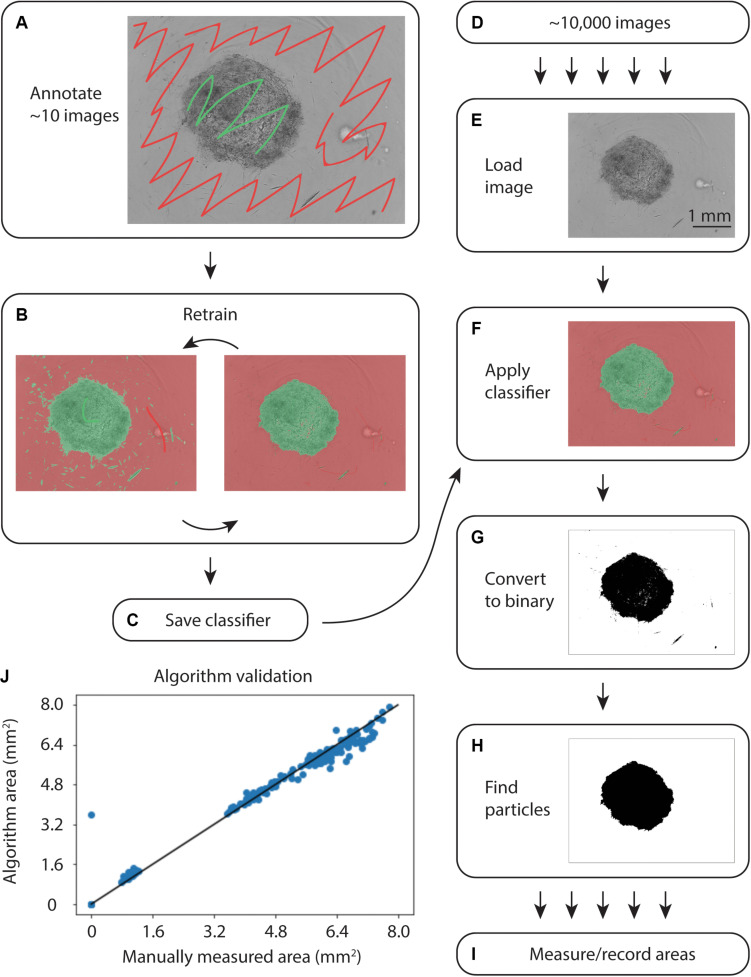
Overview of WEKA segmentation. **(A)** First, the training set of ∼10 images is annotated, clearly indicating the collagen gel and microwell plate background. **(B)** The classifier is retrained until it performs adequately. **(C)** After ensuring appropriate performance, the classifier is saved. **(D)** Approximately 10,000 images are acquired from the Incucyte microscopy system. **(E)** Images are loaded into FIJI and **(F)** the classifier created in **(A–C)** is applied. **(G)** The images are converted to binary and **(H)** particles are selected. **(I)** Finally, the area of each gel is measured and recorded. **(J)** Correlation plot of WEKA Segmentation and manual area annotation, showing a linear relationship.

To quantify collagen microgel area, we initially examined the built-in Incucyte segmentation software. However, the software performed poorly with microplate imperfections (i.e., – plate scratches) and gels at early time points, when they are relatively translucent (data not shown). We next examined WEKA Trainable Segmentation, a machine learning plugin included in the open source image processing program, FIJI (ImageJ). Figure 2 shows the process for training the WEKA Segmentation classifier. After loading an image sequence into FIJI and selecting Trainable WEKA Segmentation, the background area is manually identified with the cursor and marked as Class 1. Gel areas are similarly marked as Class 2. After training a classifier, additional annotations can be added to revise the classifier until it performs adequately. Once a satisfactory classifier has been found, it is saved for future use (Figures 2A–C).

To analyze large sets of images exported from the Incucyte in-incubator microscope, a FIJI plugin was written (Supplementary File). This plugin uses the built-in Trainable WEKA Segmentation plugin to apply the saved classifier to each image in a directory, as in Figures 2D–I. Following classification, the image was converted to binary and the largest particle was found using the built-in particles function. Areas and metadata were then recorded.

During initial testing on an Intel^TM^ Core^®^ i7-7700 CPU at 3.60 GHz, classification of a single image took 15 s. Further exploration revealed that smaller training sets enabled faster classification. The iterative training process in Figure 2 allowed for fine-tuning small training sets to achieve accurate segmentation without sacrificing speed.

To validate the automated image analysis algorithm, 300 images of microgels from early and late time points were manually annotated for comparison of area measurements. A strong correlation of 0.990 (Pearsons) with manual area measurements was achieved (Figure 2J).

### Collagen Microgel Contractions Kinetics

**FIGURE 3:**
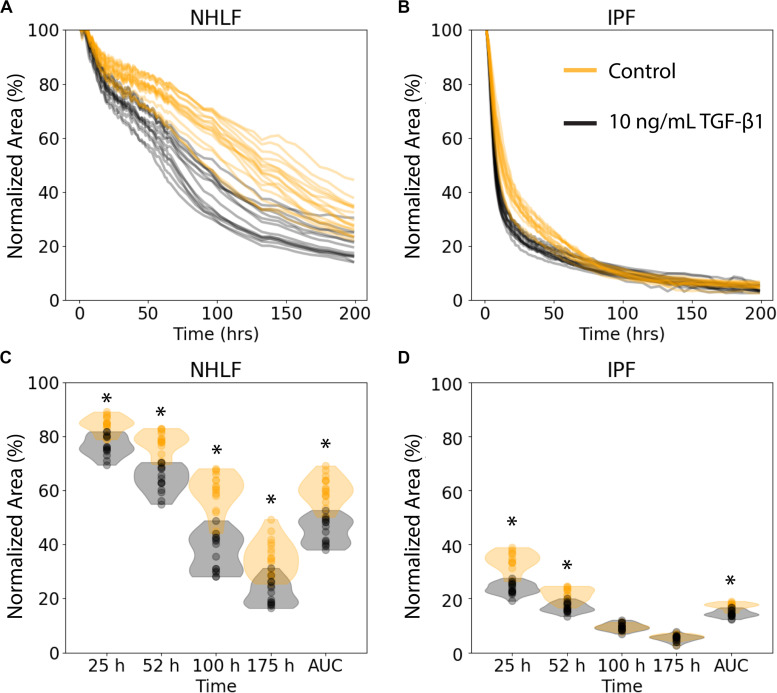
Collagen microgel contraction kinetics and use of an aggregate statistic to distinguish contraction behavior depending on imaging timepoint. **(A)** Normalized area of collagen gels over time for NHLF and **(B)** IPF cells. Each line represents an individual gel. Orange curves are for control samples, and black curves are for cells stimulated with 10 ng/ml TFG-β1. **(C)** Contraction of NHLF and **(D)** IPF cells in response to stimulation with 10 ng/mL TGF-β1. The ability to discern a difference in response depends on the timepoint at which measurements are made. However, the area under the curve more readily and consistently reflects these differences. *n* = 15 per condition. *p < 0.05 between the +/− TGF-β1 conditions on multiple *t*-tests with a Bonferroni correction.

We examined the performance of the ATPS collagen microgel contraction assay with NHLF and IPF responses to TGF-β1 stimulation at a high dose (10 ng/mL) using our automated seeding, washing, and image processing system. The NHLF had both slower baseline contraction and slower TGF-β1 activated contraction compared to IPF, as expected. However, we only tested cells from one patient of each category, so conclusions about biological differences between patients are not justified from this study alone. TGF-β1 induced a moderate increase in the rate of contraction for both cell types, as shown in Figure 3. Figures 3A,B show the area of each collagen gel (NHLF and IPF, respectively) as it contracts over time, normalized to that gel’s initial area. The same data is shown again in Figures 3C,D, but with violin plots to convey the distribution. For the NHLF cells, multiple *t*-tests with Bonferroni correction indicated significant differences with a 95% confidence interval between groups (presence or absence of TGF-β1) at each of the selected time points – 25, 52, 100, and 175 h, as well as using the area under the curve aggregated data. However, the IPF cells only showed significantly different responses to TGF-β1 at the early time points (25 and 52 h) and with the area under the curve.

### Examination of Anti-fibrotic Drugs

**FIGURE 4:**
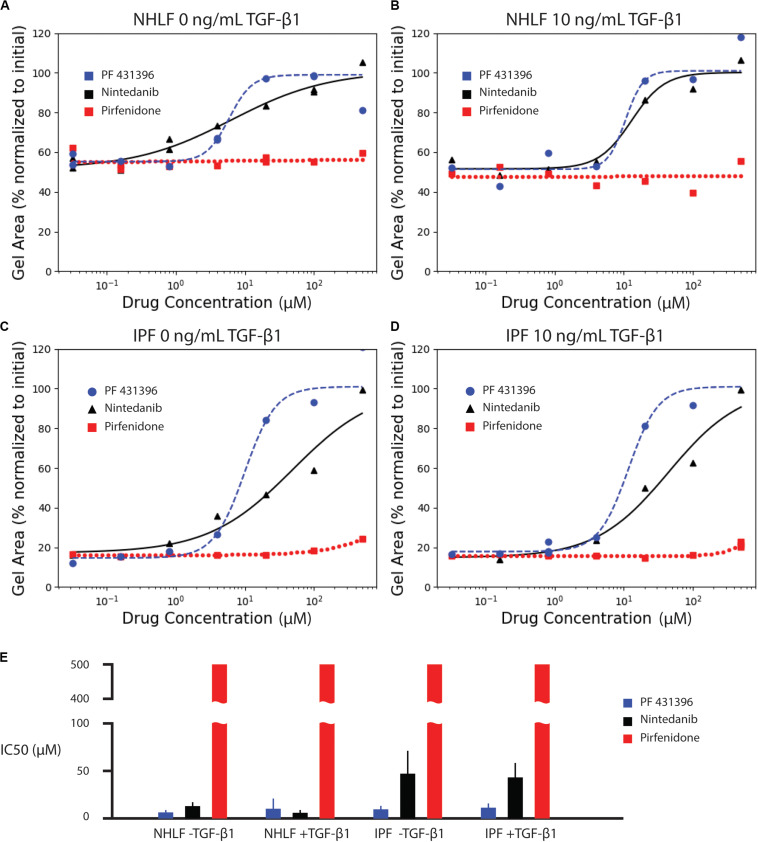
Effect of anti-fibrotic drugs (nintedanib and pirfenidone) and focal adhesion kinase inhibitor (PF 431396) on collagen contraction of NHLF cells without **(A)** and with **(B)** 10 ng/mL TGF-β1, and IPF cells without **(C)** and with **(D)** 10 ng/mL TGF-β1. The fitted parameters for IC50 are plotted in **(E)**. Error bars are the standard deviation of the parameter estimate.

We next assessed the ability of known anti-fibrotic drugs to inhibit contraction of collagen microgels. Using a concentration range of 32 nM to 500 μM, we analyzed the dynamic contraction of collagen microgels with NHLF and IPF cells, both with and without 10 ng/mL TGF-β1. For these studies, we selected the two FDA-approved fibrosis therapeutics, nintedanib (pan-kinase inhibitor) and pirfenidone (mechanism still unclear). Additionally, we examined the focal adhesion kinase (FAK) inhibitor, PF 431396 (Figures 4A–D and Supplementary Figures 1–3).

Dose response curves were fit for normalized area measurements at each time point (Supplementary Figure 4). In comparison to the high variance seen at individual time points, dose response curves for area under the curve measurements incorporated kinetic data, generating narrower standard deviations of the parameter estimate for IC50, as seen in Figure 4E. The IC50 values for each drug and cell condition are shown for AUC measurements in Figure 4E and for each individual time point in Supplementary Figure 4. The IC50 values for AUC measurements are also listed in Supplementary Table 1. Both the FAK inhibitor (PF 431396) and nintedanib showed efficacy across the cell and stimulation type, while pirfenidone did not achieve 50% effect within the range of concentrations tested.

## Discussion

In this work, we added automated image acquisition and analysis to our group’s prior development of an ATPS collagen microgel contraction assay (Moraes et al., 2013). We also analyzed normal and diseased primary human lung fibroblasts, whereas we had previously analyzed only fibroblast immortalized cell lines. Our previous work focused on the miniaturization of the ATPS collagen microgel contraction assay, opening the possibility for more effective mass transport of agonists and antagonists to the cells (Moraes et al., 2013). The addition of automated imaging and subsequent analysis in this report enabled higher throughput, as well as kinetic analysis of the collagen microgel contraction. Due to the translucent properties of the collagen microgels, the built-in image analysis software in the Incucyte was unable to accurately detect the collagen gels. This shortcoming was addressed by implementing WEKA Segmentation through a custom Jython plugin for FIJI. The WEKA Segmentation reliably yielded measurements matching those found by manually tracing the outlines of the collagen gels, indicating that the WEKA Segmentation was a sufficient tool for image processing. The automated image acquisition and analysis enabled an order of magnitude higher frequency of imaging compared to the standard daily measurement. This temporal analysis unveiled the rapid initial contraction seen in IPF-sourced fibroblasts.

For downstream analysis, it is useful to aggregate the data from an individual contraction time course into a single metric. Although some individual time points are useful to detect the increased contraction in response to TGF-β1, much of the kinetic information is lost. Therefore, we used the area under the curve to aggregate the rate of contraction for each individual gel into a single metric.

Consistent with literature reports (Jin et al., 2019), pirfenidone has little effect below 500 μM, but mildly inhibits contraction at 500 μM in all conditions (Supplementary Figure 1). Previous examinations of pirfenidone to modulate fibroblast behavior *in vitro* have required concentrations of 500 μM or higher to see statistically significant suppression of α-SMA and collagen (Nakayama et al., 2008; Conte et al., 2014). However, 500 μM was selected as the high concentration for these studies due to the requirement for high concentrations of dimethyl sulfoxide (DMSO) necessary to achieve pirfenidone concentrations above 500 μM. In this study, the DMSO concentration was kept at 0.1%. None of the concentrations of pirfenidone tested in this study produced a half maximal inhibition of contraction (Figure 4).

In contrast, nintedanib exhibits a dose-dependent inhibition of contraction in all cell conditions tested (Supplementary Figure 2). Areas of collagen microgels were normalized to their initial area. Interestingly, the inhibition of microgel contraction was largely independent of TGF-β1 for both NHLF and IPF. After a rapid initial contraction, the area reduction dramatically slowed after ∼1 day in culture for both cell types (Rangarajan et al., 2016). Our study is the only *in vitro* study to obtain IC50 values for nintedanib in NHLF and IPF cells with and without TGF-β1 in side-by-side studies. We do note that in the few *in vitro* studies that do report IC50, that those values were lower – 144 nM for inhibition of α-SMA in IPF cells (Wollin et al., 2014) and a conference abstract that notes 73 nM for inhibition of PDGF-stimulated collagen gel contraction with NHLF (Wollin et al., 2016). IC50, however, is not a fundamental constant but rather a convenient, assay-specific measure of potency. Thus, comparison of values across different experiments must be made with caution (Kalliokoski et al., 2013).

Lastly, the focal adhesion kinase (FAK) inhibitor, PF 431396, inhibited contraction at concentrations above 4 μM for all cell types, as shown in Supplementary Figure 3. Although FAK inhibition is not a widely used drug target due to many off-target effects, this experiment does corroborate previous reports indicating that NHLF contraction of collagen gels requires FAK stimulation (Liu et al., 2010; Epa et al., 2015).

These are, to our knowledge, the first reports of dose response for inhibition of TGF-β1 stimulated collagen gel contraction of primary human fibroblasts by pirfenidone, nintedanib, and PF 431396. While these results nicely demonstrate the technical capabilities, this proof-of-concept drug comparison study is limited with regards to biological conclusions by the small number of replicates and cells from just two donors.

## Conclusion

We have extended the ATPS microgel contraction assay with live-cell imaging to uncover differential phenotypic behavior of primary cells, whereas our previous methods were limited to cell lines. Because contraction is a time-dependent process, a higher sampling frequency (e.g., every hour vs. the more common every 12–24 h) can provide richer information. We assessed and optimized an automated image segmentation algorithm using WEKA machine learning to measure the areas of 10,000 collagen gel images with high temporal resolution. The assay provides added convenience and throughput, making it appropriate for secondary screening assays and dose response studies. Lastly, we report dose response characteristics for two FDA approved drugs: nintedanib, pirfenidone, as well as the FAK inhibitor, PF 431396 with healthy and diseased primary human fibroblasts, each with and without TGF-β1 activation. The calculated IC50 values confirm previous reports of lower potency for pirfenidone relative to nintedanib. This assay could provide useful phenotypic data to aid secondary and tertiary drug screens, as well as high-throughput information about primary cell behavior in basic research on fibroblast contraction.

## Data Availability Statement

The raw data supporting the conclusions of this article will be made available by the authors, without undue reservation.

## Author Contributions

CY and ST conceptualized the project idea. CY performed experiments. All authors contributed to writing the manuscript.

## Conflict of Interest

The authors declare that the research was conducted in the absence of any commercial or financial relationships that could be construed as a potential conflict of interest.
