# Supplementary material for: Kinetic Analysis of Label-Free Microscale Collagen Gel Contraction Using Machine Learning-Aided Image Analysis
Source: Front Bioeng Biotechnol. 2020 Sep 22;8:582602. doi: 10.3389/fbioe.2020.582602 (PMC7537788; doi:10.3389/fbioe.2020.582602)
Supplement: Supplementary file 3 [file Table_1.docx]

Supplementary Materials

# Supplementary Figures


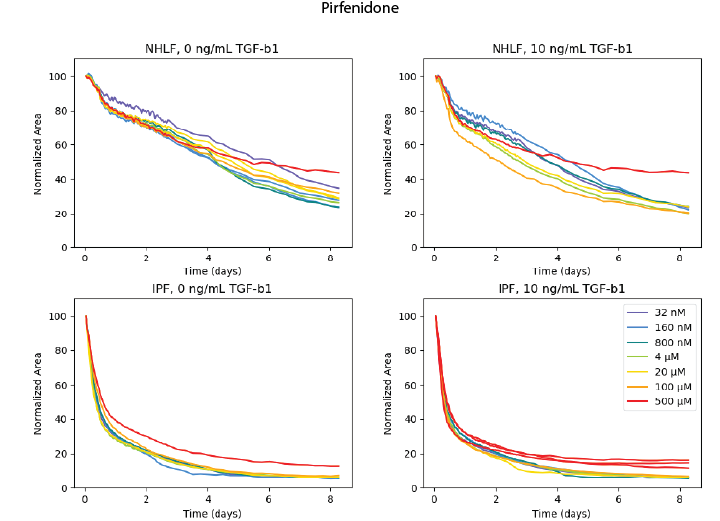


**Supplementary Figure 1.** Contraction of primary human fibroblasts with addition of pirfenidone. Areas are normalized to the initial area of each microgel. N=1-2 per condition.


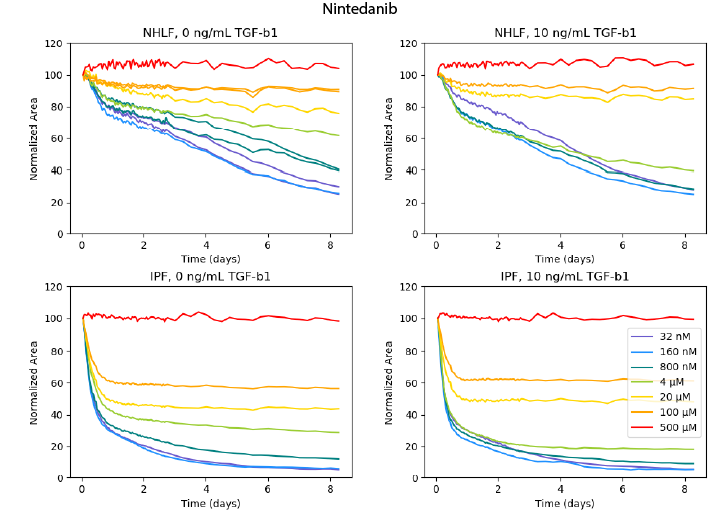


**Supplementary Figure 2.** Contraction of collagen microgels by primary human lung fibroblasts with addition of nintedanib. Areas of collagen microgels were normalized to their initial area. N=1-2 per condition.


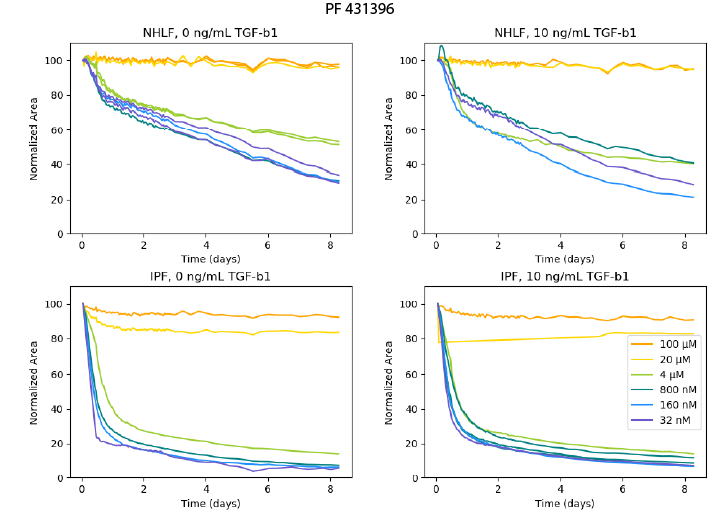


**Supplementary Figure 3.** Contraction of collagen microgels with primary human lung fibroblasts and the FAK inhibitor, PF 431396. Areas are normalized to the initial area of each microgel. N=1-2 per condition.

**
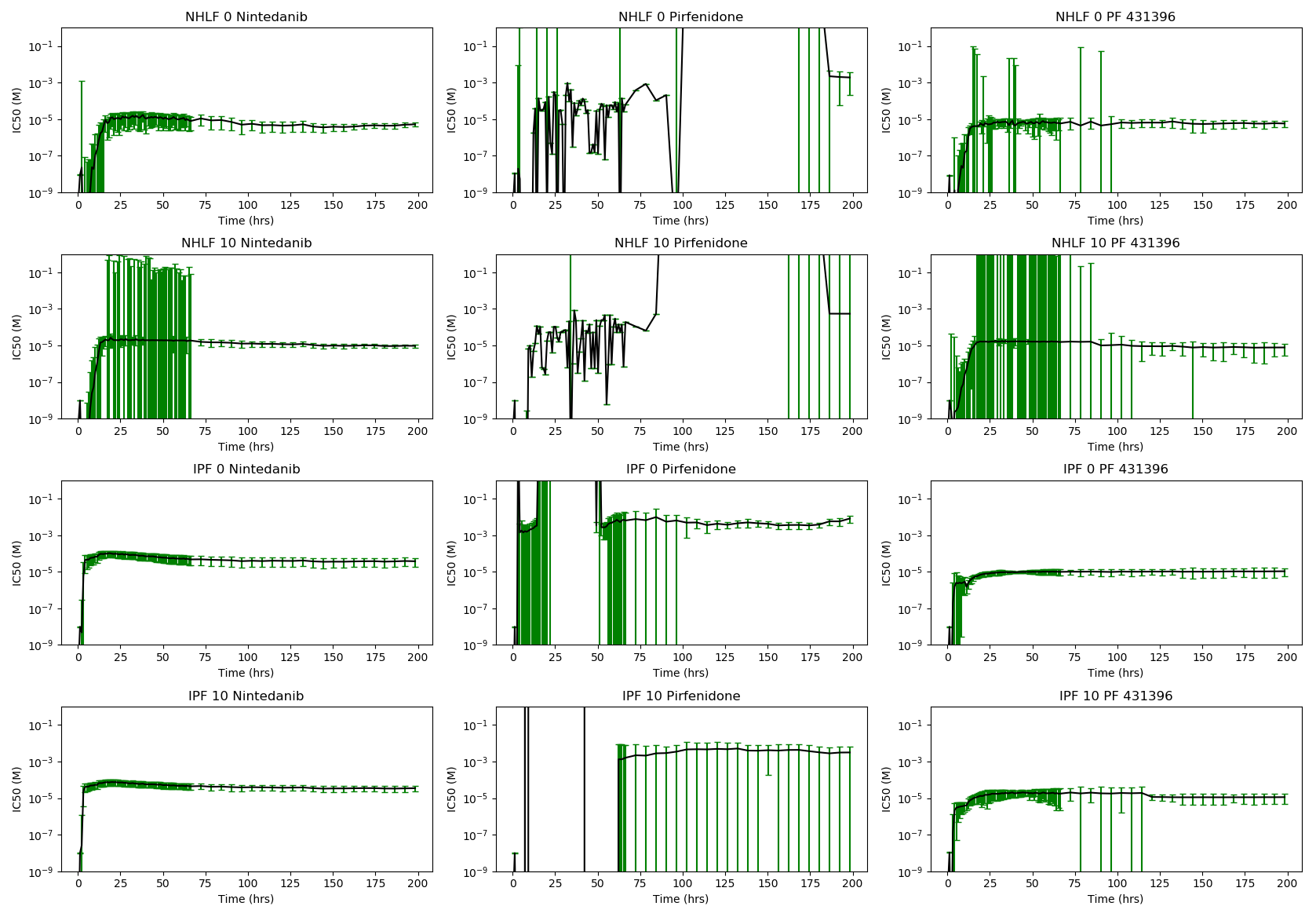
Supplementary Figure 4.** Fitted values for IC50 for each cell and TGF-β1 combination, graphed over individual time points. Error bars are standard deviation, calculated from the model fit covariance.

**Supplementary Table 1.** Fitted IC50 values for cell, stimulant, and drug combinations.

|  | **NHLF IC50 (μM)** | |  | **IPF IC50 (μM)** | |
| --- | --- | --- | --- | --- | --- |
| **Drug** | **-TGF-β1** | **+TGF-β1** |  | **-TGF-β1** | **+TGF-β1** |
| PF 431396 | 6.3$\pm$2.5 | 10.2$\pm$10.7 |  | 9.8$\pm$ 3.6 | 11.5$\pm$4.3 |
| Nintedanib | 5.9$\pm$3.0 | 12.9$\pm$ 4.1 |  | 47.1$\pm$23.9 | 43.1$\pm$15.2 |
| Pirfenidone | >500.0 | >500.0 |  | >500.0 | >500.0 |
